# Recent advances and applications of human brain models

**DOI:** 10.3389/fncir.2024.1453958

**Published:** 2024-08-05

**Authors:** Kaneyasu Nishimura, Hironobu Osaki, Kotaro Tezuka, Daisuke Nakashima, Shintaro Numata, Yoshito Masamizu

**Affiliations:** Laboratory of Functional Brain Circuit Construction, Graduate School of Brain Science, Doshisha University, Kyotanabe, Japan

**Keywords:** human pluripotent stem cells, brain region-specific neurons, brain organoids, assembloids, disease modeling, bioengineering

## Abstract

Recent advances in human pluripotent stem cell (hPSC) technologies have prompted the emergence of new research fields and applications for human neurons and brain organoids. Brain organoids have gained attention as an *in vitro* model system that recapitulates the higher structure, cellular diversity and function of the brain to explore brain development, disease modeling, drug screening, and regenerative medicine. This progress has been accelerated by abundant interactions of brain organoid technology with various research fields. A cross-disciplinary approach with human brain organoid technology offers a higher-ordered advance for more accurately understanding the human brain. In this review, we summarize the status of neural induction in two- and three-dimensional culture systems from hPSCs and the modeling of neurodegenerative diseases using brain organoids. We also highlight the latest bioengineered technologies for the assembly of spatially higher-ordered neural tissues and prospects of brain organoid technology toward the understanding of the potential and abilities of the human brain.

## Introduction

The study of the human brain, including neural circuit formation, brain development, and physiological function is an interesting but unexplored research field. Many studies have attempted to clarify these topics using *in vitro* and *in vivo* model organisms such as cell lines and animals because of the inaccessibility of human brain tissue. For decades, the *in vitro* culture of primary neurons dissected from rodent brains has been an indispensable experimental tool to investigate neural circuit formation and their physiology and function. Although numerous findings have relied on primary animal neurons, the cellular architectures of neurons in animals markedly differ from those in humans and produce irreconcilable results among species. Similarly, various human neural cell lines including SH-SY 5Y (neuroblastoma), IMR-32 (neuroblastoma), and LUHMES cells (immortalized neural cell line) have been used as *in vitro* models of human neurons to examine cellular phenotypes (Reynolds and Pérez-Polo, 1975; Biedler et al., 1978; Lotharius et al., 2002). These cell lines can proliferate infinitely under culture conditions but exhibit insufficient neuronal maturation and functions. Thus, these models are unsatisfactory for exploring the phenotype of human neurons. To solve these issues, human pluripotent stem cell (hPSC)–derived neurons are highlighted as a new experimental model to provide a more accurate understanding of the human brain. Recently, stem cell technology has been used to produce brain organoids as a remarkable human brain model that has a three-dimensional (3D) structure that resembles the trajectory of brain development (Sasai, 2013). Brain organoids are a promising tool to study the development, neural circuit formation, and physiological function of the brain in healthy and pathological conditions.

In this review, we take an overview of neural differentiation technologies based on hPSCs and disease modeling of neurodegenerative disorders using brain organoid technology. We further summarize the latest engineered technologies to generate spatially higher-ordered neural tissues and their applications.

## Generation of human neurons from hPSCs

Human embryonic stem cells (hESCs) were first established from blastocysts in 1998 (Thomson et al., 1998). hESCs can give rise to various somatic cells including neurons and provide a research platform to investigate human neurons *in vitro*. However, the use of hESCs is strictly regulated because of ethical concerns in some countries as hESCs originate from human embryos. In 2007, human induced pluripotent stem cells (hiPSCs) were developed from fibroblasts by introducing four transcription factors (Takahashi et al., 2007; Yu et al., 2007). hiPSCs can be generated from any individual and provide autologous transplantation, personalized medicine, and disease modeling in the respective patients by inheriting their genetic background. Furthermore, the culture conditions of hPSCs have been improved to achieve chemically defined (Chen et al., 2011) and feeder-free monolayer conditions (Nakagawa et al., 2014; Rodin et al., 2014). The improved culture conditions enable reproducible and scalable outcomes and realize the clinical application of hPSC-derived neurons. In parallel, derivation methods of hPSC-derived neurons have been developed. Highly efficient neural induction methods have been established by using chemical inhibitors of the SMAD-signaling pathway that is important for forming the mesoderm and endoderm, which sufficiently promotes cell fate determination into neuroectoderms (Chambers et al., 2009; Morizane et al., 2011). Subsequent studies have shown that various brain regions including the cerebral cortex (Kadoshima et al., 2013; Imaizumi et al., 2015; Qi et al., 2017), basal ganglia (Amimoto et al., 2021; Conforti et al., 2022; Hunt et al., 2023), hypothalamus (Zimmer et al., 2016; Kasai et al., 2020), midbrain (Doi et al., 2014; Kirkeby et al., 2017; Kim et al., 2021; Nishimura et al., 2023), cerebellum (Muguruma et al., 2015; Nayler et al., 2021), and spinal cords (Lippmann et al., 2015; Ogura et al., 2018) were induced from hPSCs by small molecules and patterning factors that recapitulate the process of brain pattern formation during development. The long-term maturation culture of hPSC-derived neurons was subsequently achieved to form more complex neural networks that exhibit spontaneous neuronal activities that can be recorded by electrophysiological analysis and calcium imaging (Odawara et al., 2016; Zabolocki et al., 2020). These recording technologies can be used to investigate the phenotype of neurological diseases *in vitro* (Virdi et al., 2022; Hussein et al., 2023).

Two-dimensional (2D) monolayer culture has been predominately used for the *in vitro* culture of hPSC-derived neurons to investigate neural induction, neural circuit formation, and neuronal activities. 2D-cultured neurons randomly extend their dendrites and axons on extracellular matrix coatings and then form functional synapses with the other neurons in a culture dish. The neuronal properties of hPSC-derived neurons provide an *in vitro* model system that represents neurophysiological and pathophysiological features to study the phenotype of neurological diseases and screen new drug candidates to treat neurological diseases. Although 2D-culture systems offer a simple handling, reproducible, and cost-effective method to observe neuronal aspects, neurons are randomly formed, and their neural circuits lack the topology of the *in vivo* brain (Castillo Ransanz et al., 2022). To overcome these limitations, recent research has shifted toward 3D cell culture such as spheroids and organoids to challenge the assembly of brain and neural circuit formation. hPSC suspensions spontaneously form 3D embryoid bodies (EBs) in free floating culture systems and are applicable as *in vitro* differentiation models into three-germ layers under conditions lacking basic fibroblast growth factor (bFGF) (Itskovitz-Eldor et al., 2000). By modifying EB methods, Eiraku et al. (2008) developed a serum-free floating culture of EB-like aggregates with the quick reaggregation (SFEBq) method. This method involves seeding single-cell dissociated PSCs in wells of U-shaped 96-well plates to quickly obtain uniformed cell aggregates. The hESC-derived aggregates formed by the SFEBq method spontaneously differentiate into neuroectoderm and form self-organized cortical layers with histological signatures that resemble the early cellular architecture seen during cortical development (Kadoshima et al., 2013). This advanced stem cell culture method provided an important breakthrough toward understanding the self-organized tissue formation of the human brain. From this point, various brain region-specific neurospheres were induced from hPSCs based on SFEBq methods for specified brain regions by treatment with patterning factors and equivalent small compounds (Kikuchi et al., 2011; Muguruma et al., 2015; Sakaguchi et al., 2015; Ozone et al., 2016). In 2013, Lancaster et al. (2013) developed a differentiation method of cerebral organoids from hiPSCs of a patient with microcephaly, a neurodevelopmental disorder. In this method, neurospheres were embedded in Matrigel droplets at an early stage of the induction and then cultured in a spinning bioreactor without any patterning factors. Brain organoids spontaneously formed a self-organized cortical structure that mimicked the cellular aspects of cortical development. The brain organoid technology recaptures the spontaneous orientation of positional information to form the morphology of the *in vivo* brain by mimicking multicellular aspects with complex cell-to-cell interactions and dynamics during brain development.

## Disease modeling using hiPSC-derived brain organoids

**Figure 1:**
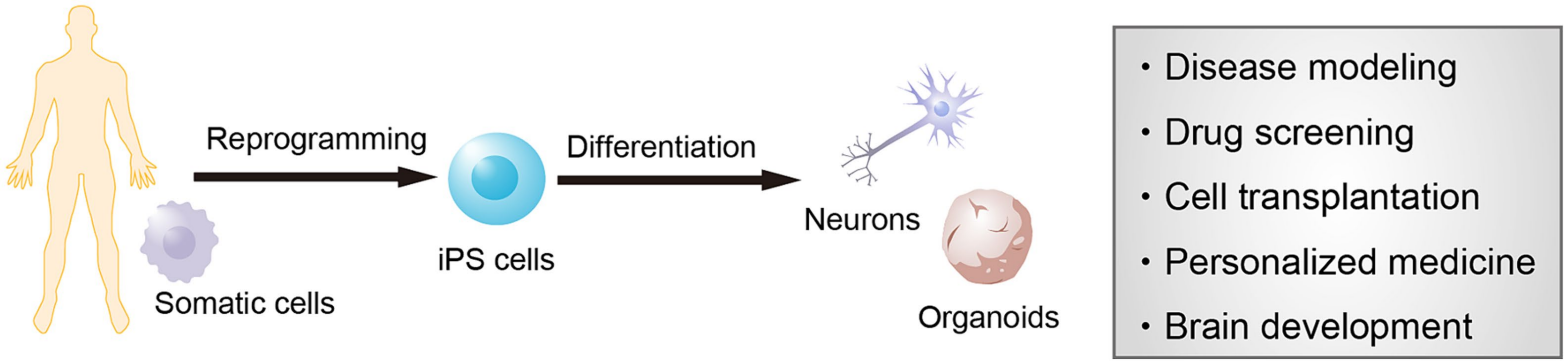
Modeling of neurological diseases using human induced pluripotent stem cells (hiPSCs). iPSCs are established from the somatic cells of any individual and provide neural cells and brain organoids. These cells can be used for modeling the pathological features of patients as well as for screening new drug candidates, cell transplantation therapy, personalized medicine, and brain development.

Most of our understanding of the pathological features of neurodegenerative diseases, including Alzheimer’s disease (AD) and Parkinson’s disease (PD), has been derived from postmortem studies of patients. Although the accurate observation of the brain pathology in postmortem biopsy samples has offered definitive diagnoses, investigating cellular dynamics accompanied by disease progression and cellular phenotypes of the living neurons remains challenging. Consequently, the molecular mechanisms underlying the onset and progression of the disease are not fully understood and adequate treatments to overcome them remain lacking. An alternative strategy to understand the pathology of neurogenerative disorders is to investigate phenotypes along the onset and progression of pathological symptoms using brain organoids derived from patients (Figure 1). hiPSC-derived brain organoids provide an appropriate approach for observing cellular phenotypes of late-onset neurodegenerative diseases. Disease-specific hiPSCs generated from refractory diseases inherit the genetic background of the donor somatic cells, and disease-specific hiPSC-derived brain organoids are therefore useful for studying the disease mechanisms and screening drugs in both idiopathic and genetic diseases. In particular, recapturing abnormalities in protein aggregation, neuroinflammation, and neural function that underlies the pathogenic process before the onset of symptoms provide important findings in investigating new therapeutic strategies and preventative medicine.

AD is the most common neurogenerative disorder in our aging society (Blennow et al., 2006). Pathological features of patients with AD are the deposition of amyloid plaques and neurofibrillary tangles that are linked to the accumulation of amyloid-β (Aβ) protein and hyperphosphorylated tau protein, respectively. This abnormal protein metabolism causes neuroinflammation and neurodegeneration in the brain before the onset of clinical symptoms, leading to progressive cognitive impairments. The brain organoids has been used to recapture the pathological features of familial AD carrying the mutations of presenilin 1 and/or amyloid precursor protein in various reports (Raja et al., 2016; Gonzalez et al., 2018; Choe et al., 2024). The authors observed Aβ accumulations and phosphorylated tau protein in brain organoids of patients with familial AD that appear from the early stage of AD. Furthermore, Shimada et al. (2022) generated forebrain organoids from patients with familial AD and observed Aβ pathology and an increased presence of phosphorylated tau protein in the organoids, which is a hallmark of the pathology of AD; tau fibrils were also observed in cell bodies and the neurites of organoids that were injected with adeno-associated virus-expressing mutant tau (P301L). These experimental models recapture the pathogenic process of Aβ deposition and tauopathology of AD.

PD is a second common neurogenerative disorder that is characterized by the selective death of dopaminergic neurons in the substantia nigra pars compacta (Kalia and Lang, 2015). The major pathological feature is the accumulation of α-synuclein protein in the Lewy bodies (LBs) found in the remaining dopaminergic neurons of patients with PD (Spillantini et al., 1997). Either missense mutations or copy number variations in the *SNCA* gene have been reported, and successfully generated iPSCs from patients have been used to reveal the pathological features of the disease using patient iPSC-derived midbrain organoids containing dopaminergic neurons. Mohamed et al. (2021) demonstrated that midbrain organoids generated from PD-iPSCs carrying an *SNCA* triplication recapitulate key synucleinopathy features such as the accumulation of α-synuclein aggregates and the degeneration of dopaminergic neurons. Jo et al. (2021) investigated midbrain organoids derived from hiPSCs carrying mutations in both the glucocerebrosidase and *SNCA* genes. LB-like inclusions accumulated in the organoids, which exhibited cell death of dopaminergic neurons, resembling the pathology observed in patients with PD. Collectively, human brain organoid models can emphasize the significance of modeling neurodegenerative disorders, offering more accurate representations of disease pathology and facilitating the investigation of new therapeutic strategies and drug screening.

## Advanced technology in the generation of multiregional brain organoids

**Figure 2:**
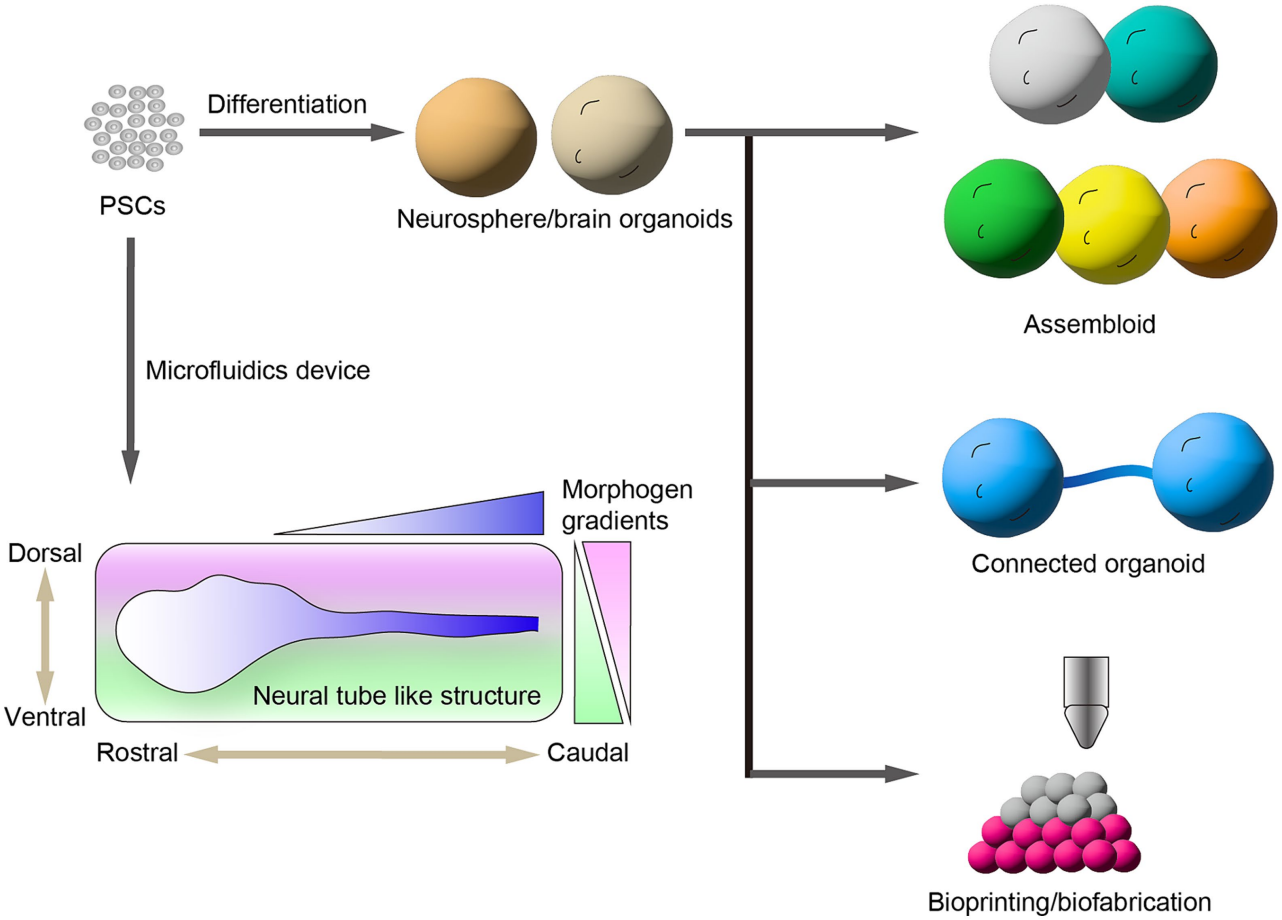
Generation of brain organoids from human pluripotent stem cells (hPSCs). hPSCs provide brain region-specific neurospheres and organoids. Assembloids are generated by the fusion of multiple brain organoids that have different brain regions. Recent bioengineering technologies provide connected organoids and spatially arranged 3D neural tissues. Microfluidic devices control the morphogen gradient to induce the formation of neural tube-like structures along the rostral–caudal and dorsal–ventral axes.

Region-specific brain organoids can be used as appropriate tools to investigate specific subtypes of neurons because most neurological diseases are primarily lesion-specific subtypes of neurons corresponding to distinct brain regions. By advancing region-specific brain organoids, multiregional brain organoids have been generated to investigate the neural circuit formation, neuron–neuron interaction, and pathology across brain regions (Figure 2). The formation of brain regions is substantially controlled by orchestrated activities of morphogens including WNT, sonic hedgehog (SHH), and bFGF along the rostral–caudal and dorsal–ventral axes during brain development. Brain region-specific organoids are generated by resembling the corresponding signaling pathway by treatment with morphogens and/or their equivalent small molecules during organoid induction from hPSCs (Imaizumi et al., 2015). Recently, Pavon et al. (2024) generated spatially patterned forebrain organoids from hESCs using an engineered microdevice. They recaptured the SHH gradient to form the dorsal–ventral axis in forebrain organoids that was regionalized from the cortex to basal ganglia of the forebrain along the dorsal–ventral axis. Rifes et al. (2020) reported the formation of neural tubes from hESCs by mimicking the gradient activation of a morphogen in a microfluidics device, named microfluidic-controlled stem cell regionalization (MiSTR). This method recaptures the WNT-activating gradient that crucially controls the rostral–caudal axis formation in neural tube formation. The neural tissue generated by MiSTR was regionalized along with rostral–caudal patterning from the forebrain and midbrain to the hindbrain. Alternative approaches have formed microfluidic neural tube-like structures (μNTLSs) that have a brain-to-spinal cord along the rostral–caudal and dorsal–ventral axes from hiPSCs (Xue et al., 2024). μNTLSs were produced using a microfluidics device that first produced a patterned rostral–caudal axis and then a dorsal–ventral axis by mimicking the morphogen gradients. These approaches provide a proof of concept to generate multiregional neural tissues from hPSCs by controlling the morphogen gradients.

Another approach to generating multiregional brain organoids from hPSCs is to assemble multiple brain organoids that have different regions. The different regional specified brain organoids were cocultured for fusion as assembloids. Assembloids have significant advantages for studying the inter-regional neural circuit formation. Birey et al. (2017) reported the generation of hiPSC-derived assembloids containing pallium and subpallium that recapitulate the saltatory migration of interneurons generated in the subpallium to the pallium region. Using this model, the authors found the interneurons induced from hiPSCs of patients with Timothy syndrome, a developmental disorder that exhibits abnormal saltatory migration and revealed their molecular mechanisms (Birey et al., 2022). Similarly, hPSC-derived assembloid models were used to investigate the formation of inter-regional neural networks among brain regions such as the cortico–striatal pathway (Miura et al., 2020), retinofugal projection (Fligor et al., 2021), and striatal–midbrain pathway (Ozgun et al., 2024; Wu et al., 2024). Furthermore, Andersen et al. (2020) generated multiregional assembloids fusing the three organoids including the cerebral cortex, spinal cord, and skeletal muscle that exhibit muscle contraction by responding to the optogenetic stimulation of cortical neurons via motor neurons. Reumann et al. (2023) generated spatially arranged ventra–midbrain–striatum–cortical organoids (MISCOs) from hPSCs to study the functional innervation of dopaminergic neural circuits into either the striatum or cortex. Moreover, the effects of chronic cocaine treatment on MISCOs were studied to show that dopaminergic axons innervated in either the striatum or cortex were perturbed by cocaine treatment. Interestingly, the authors injected dopaminergic progenitors into the ventral midbrain region of MISCOs and observed the reconstruction of dopaminergic neural circuits into the striatum and cortex regions. This model system is a useful platform to estimate the cellular aspects and maturation of grafted dopaminergic neurons by the recapitulation of cell transplantation therapy for patients with PD.

Recently, innovative bioengineering technologies such as bioprinting and biofabrication have been used to generate spatially arranged 3D neural tissues from hPSCs. These bioengineered technologies allow a more accurate assembly of neural tissues by precisely controlled manipulations. Roth et al. (2023) developed a magnetic-based bioprinting approach, termed spatially patterned organoid transfer (SPOT). This involved embedding dorsal and ventral cortical organoids induced from hiPSCs in an iron nanoparticle-laden scaffold to construct a spatially controlled patterning by using a magnetized 3D printer. Using SPOT, the authors generated precisely arranged neural assembloids and glioma organoids. Jin et al. (2023) fabricated a 3D neural tissue of a cortical column derived from hiPSCs using a droplet-based 3D printing technology. The authors induced upper- and deep-layer cortical progenitors from hiPSCs and printed a spatially controlled layer structure resembling the cerebral cortex. Furthermore, the 3D cortical tissue implanted into *ex vivo* mouse cerebral cortex projected neurites into the mouse brain and exhibited an inter-regional correlation of spontaneous calcium oscillation corresponding to neuronal activities.

Although the emerging approaches have generated multiregional neural tissues and organoids, regional neural circuits can spontaneously but randomly connect to each other across the brain region. Another approach investigated the reciprocally axonal connection of brain organoids such as motor nerve organoids (Kawada et al., 2017) and cortical organoids (Osaki et al., 2024), in which axonal extension was guided in a thin microchannel of a microfluidic device. Using the microfluidic device, Martins-Costa et al. (2024) produced the corpus callosum (CC)-like tracts to investigate the impaired formation of long-range axons of patients with agenesis of the CC-carrying *ARID1B* mutations. These advanced approaches for multiregional brain tissues and organoids provide more accurate brain models for understanding brain development, neural circuit formation, and phenotypes of neurological diseases across brain regions.

## Prospects

Most neural differentiation methods have been established using 2D culture systems owing to a simple handling and stable outcome, rather than 3D culture systems. Because of these advantages, hPSC-derived neurons have been used for clinical applications in drug repositioning and cell transplantation therapy for neurological diseases (Kondo et al., 2017; Fujimori et al., 2018; Doi et al., 2020; Piao et al., 2021). In addition, 2D cultured hiPSC-derived neurons have recently become commercially available, allowing researchers to directly investigate their research using human neurons without the need of differentiation experiments. These manufactured products provide research advantages for understanding and using human neurons.

Brain organoid technology offers an innovative approach to gaining a deeper understanding of the cellular architectures of human brain structure and physiology *in vitro*, which is difficult to explore using other model organisms, such as cell lines and animals. These human brain models recapitulate the pathological features of neurological diseases at morphological, molecular, and genetic levels and can be applied for drug screening. However, most brain organoid models mainly contain neural lineages such as neural stem cells, neurons, astrocytes and oligodendrocytes because hPSCs are specified into neuroectodermal lineages along the differentiation. Consequently, most brain organoids lack fundamental cell types such as microglia and blood vessels found in the brain. Future studies would assemble more sufficient brain models to capture the multicellular dynamics and structures found in the brain by including these cell types together with neural lineages (Kong et al., 2023; Park et al., 2023; Takata et al., 2023; Dao et al., 2024; Shaji et al., 2024). The functional properties of brain organoids are important to understand to ensure that these are equivalent to those of the brain. One approach to assuming the functional properties of brain organoids is to observe the neural activities after their implantation in the brain. Observing the synchroneity and correlation of brain organoids with the brain activity of the recipient would be an effective approach to reveal the functional properties of these organoids. Indeed, hPSC-derived cortical organoids have survived in the cerebral cortex of the rat brain following implantation and subsequently elongated to the brainstem and spinal cord along to corticospinal tract (Kitahara et al., 2020; Cao et al., 2023). Furthermore, recording technologies such as fiber photometry and two-photon calcium imaging have revealed synchronous neural activities of brain organoids following implantation into the animal brain (Revah et al., 2022). The combination of brain organoid research with imaging and recording technologies supports the investigation of the neuronal activities of brain organoids and is also applicable to estimating their similarity to those of the brain.

Similarly, recent progress of hPSC-derived neurons and brain organoids are highlighted by fruitful collaborations with numerous advanced technologies such as genome editing, transcriptome analysis, soft nanomaterials, and machine learning. The collaboration of bioengineered neural networks such as micropatterned neural networks and brain microphysiological systems has particularly enabled the investigation of biological computing and biological intelligence (Kagan et al., 2022; Metzger et al., 2022; Yamamoto et al., 2023). The cross-disciplinary scientific trends with hPSC-derived neurons and brain organoids would lead the higher-ordered innovation for a more accurate understanding of the potential and capacities of the human brain.

## Author contributions

KN: Conceptualization, Writing – original draft. HO: Supervision, Writing – review & editing. KT: Visualization, Writing – review & editing. DN: Visualization, Writing – review & editing. SN: Visualization, Writing – review & editing. YM: Supervision, Writing – review & editing.
